# Oocyte Maturation and miRNAs: Studying a Complicate Interaction to Reveal Possible Biomarkers for Female Infertility

**DOI:** 10.3390/diseases12060121

**Published:** 2024-06-04

**Authors:** Eleni Nazou, Anastasios Potiris, Despoina Mavrogianni, Eirini Drakaki, Aris-Anargyros Vogiatzis, Vaia Sarli, Tereza Vrantza, Athanasios Zikopoulos, Konstantinos Louis, Chara Skentou, Periklis Panagopoulos, Peter Drakakis, Sofoklis Stavros

**Affiliations:** 1 Third Department of Obstetrics and Gynecology, University General Hospital “ATTIKON”, Medical School, National and Kapodistrian University of Athens, 124 62 Athens, Greece; elenaz@med.uoa.gr (E.N.); arisvog@gmail.com (A.-A.V.); vanasarli@yahoo.gr (V.S.); terezavrantza@hotmail.com (T.V.); thanzik92@gmail.com (A.Z.); kostaslouisss@gmail.com (K.L.); perpanag@med.uoa.gr (P.P.); pdrakakis@med.uoa.gr (P.D.); sfstavrou@med.uoa.gr (S.S.); 2 First Department of Obstetrics and Gynecology, Alexandra Hospital, Medical School, National and Kapodistrian University of Athens, 115 28 Athens, Greece; dmavrogianni@med.uoa.gr (D.M.); eirinidrak@med.uoa.gr (E.D.); 3 Department of Obstetrics and Gynecology, Medical School, University of Ioannina, 45110 Ioannina, Greece; haraskentou@uoi.gr

**Keywords:** microRNAs (miRNAs), infertility, granulosa cells, cumulus cells, Dicer

## Abstract

Cellular metabolism, apoptosis, fertilization, and proliferation of granulosa cells belong to a battery of processes where microRNAs can be detected and associated with infertility. The aim of the present review is to focus on mammalian oocyte maturation events and the association between oocyte growth and miRNA expression. PubMed/Medline, Google Scholar and Scopus databases were searched, and 33 studies were included. Regarding the correlation among miRNA expression and the regulation of granulosa cells and cumulus cells, the most important miRNAs were let-7b, let-7c and miR-21. Additionally, the loss of Dicer, an enzyme involved in miRNA biogenesis, is probably a crucial factor in oogenesis, oocyte maturation and embryogenesis. Furthermore, miRNAs interfere with different cellular mechanisms like apoptosis, steroidogenesis, genome integrity, angiogenesis, antioxidative response and, consequently, oocyte maturation. Hence, it is of major importance to clarify the role and mechanism of each miRNA as understanding its action may develop new tools and establish new diagnostic and treatment approaches for infertility and ovarian disorders.

## 1. Introduction

Infertility is commonly caused by ovulatory dysfunction, male factor problems, and tubal disease. Additionally, unexplained infertility remains a setback for approximately 15% of couples [1]. Furthermore, fertility is age-related, especially for women, as maternal age plays a crucial role and is related to ovary aging [2]. Oocyte maturation, initiates with the first meiotic progression of female germ cells during embryonic development and sustains at the dictyate stage of meiotic prophase I. The imperative event of folliculogenesis begins with fetal development at 4 months of pregnancy [3]. The oocytes from meiotic prophase I, through metaphase II (MII), have high transcriptional activity. Consequently, the mRNAs and proteins that are produced support early embryogenesis [4,5]. In the phase of primordial follicles, oocytes are surrounded by squamous granulosa cells (GCs), which promote steroidogenesis [6]. When primary follicles differentiate to secondary follicles, GCs proliferate and form multiple layers [7]. GC interactions play an important role in oocyte growth, cumulus triggering, expansion and, finally, ovulation, which prepares the oocyte to be fertilized [8,9].

**Figure 1:**
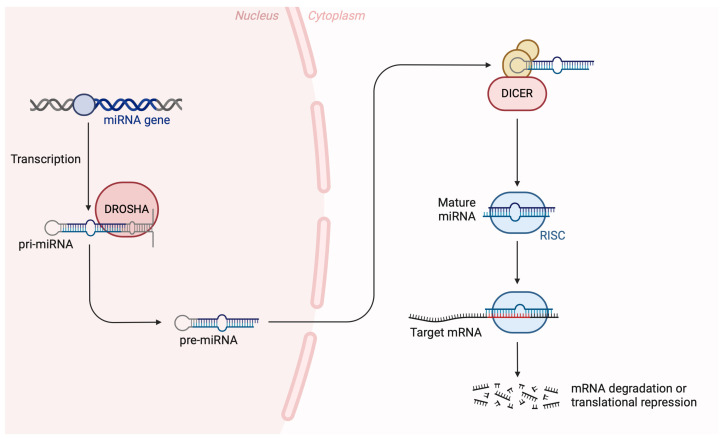
The canonical pathway of miRNA biogenesis. Precursor miRNA molecules undergo nuclear and cytoplasmic processing events, carried out by the endoribonucleases DROSHA and DICER to produce mature miRNAs that are loaded onto the RISC (RNA-induced silencing complex) to exert their biological function. Created with BioRender.com on 25 April 2024.

In recent years, many disorders have been linked to miRNA expression leading to the proposition that it may be used as a biomarker [10]. MiRNAs are small, single-stranded, and non-coding RNA molecules (19–22 nucleotides), which regulate a gene expression during post-transcriptional stage by base-pairing to mRNAs [11,12]. Approximately 1% to 5% of all genes encode miRNAs, regulating 30% of gene expression [12]. MiRNAs are produced by two RNase III proteins, Drosha and Dicer, that transcript primary into mature miRNA [13,14]. These molecules interact first with Drosha and DGCR8 in the nucleus and then are released as pre-miRNAs into the cytoplasm, cleaved by Dicer and shaping the miRNA duplex [15]. Figure 1 provides a brief illustration of the canonical pathway of miRNA biogenesis.

As miRNAs are identified in developmental timing, fertilization, early embryonic development and granulosa cell proliferation, ovaries, oocytes, cumulus cells and preimplantation embryos may express many of them [16,17,18,19,20,21]. Single point mutations of miRNA sequences, or differential expression of miRNA transcripts, may be related to various cellular mechanisms [22]. Consequently, these molecules could be responsible for disorders like endometriosis, premature ovarian failure, and polycystic ovarian syndrome by initiation of different signaling pathways [23,24].

This article focuses on the events and development related to mammalian oocyte maturation and association of oocyte growth with miRNAs expression. We will discuss the fundamental related mechanisms in order to better understand female reproduction by providing information in detail.

## 2. Materials and Methods

A comprehensive literature search was conducted using the PubMed/Medline, Google Scholar, and Scopus databases from October 2023 to February 2024 with the aim to describe the relationship between miRNAs and oocyte maturation. The search terms used included “Oocyte maturation”, “miRNAs”, “micro RNAs”, “Infertility”, “Granulosa Cells”, “Female Reproduction” and “Assisted Reproduction”. Boolean operators (OR, AND) are administered, combined with those keywords either used as presented, separately and in combination. Two independent reviewers, E.N. and A.P., assessed abstracts of the retrieved results; the content of full-text publications that were eligible was further assessed. A third reviewer, D.M., was responsible for making the decision if a study was selected by only one reviewer.

**Figure 2:**
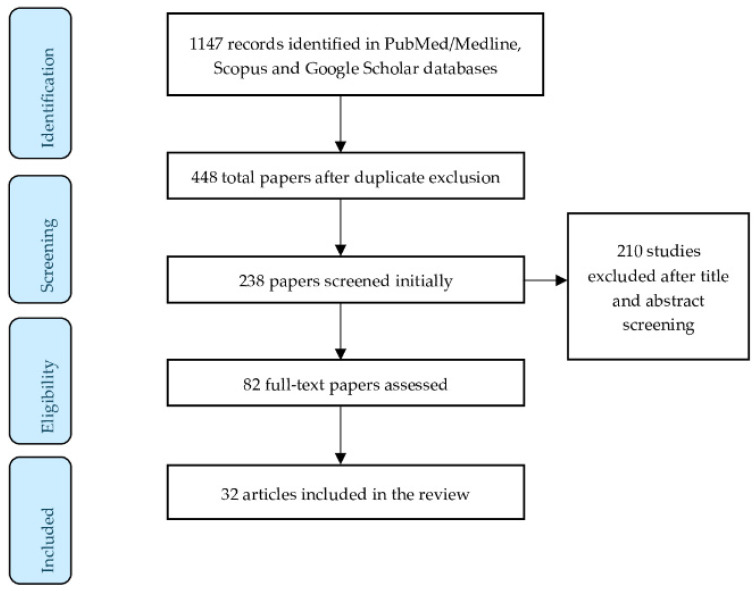
Flow diagram of the study selection process.

A total of 1147 articles were collected via different databases. In total, 82 full text articles were assessed and 32 articles/studies were suitable for providing information in this literature review. The criteria for article eligibility consist of original articles, case reports, reviews, systematic reviews, and meta-analyses. No English language written articles were excluded. Figure 2 illustrates the study selection process.

## 3. Results

Cumulus–oocyte complex (COC) contains oocyte and cumulus cells (CCs), which supply molecular nutrients and help the development of oocytes [25]. GCs and CCs are controlled by various miRNAs, enhancing the conviction that miRNAs are key factors for reproduction [26,27]. Also, microRNA expression profiling shows that miRNAs may be detected in microvesicle and exosome preparations from follicular fluid (FF) [28]. Regarding the correlation among miRNAs expression and the regulation of GC and CC, many studies have been published. Sirotkin et al. showed that inhibiting miR-15a affects the proliferation of human GCs by decreasing the proliferating cell nuclear antigen levels (PCNA) [29]. Moreno et al. examined 30 women undergoing ICSI treatment and found the expression of 866 miRNAs in FFs and GCs. The results showed 13 differentially expressed miRNAs (2 up-regulated and 11 down-regulated from MII to GV oocytes). Also, the comparison between MII and MI revealed seven differentially expressed miRNAs in MII (three of them up-regulated and four down-regulated). Only one, hsa-miR-424, was detected in higher levels in women of advanced age [30]. Additionally, Santonocito et al. described the up-regulation of hsa-miR-483 and hsa-miR-339 in FF, which in the study of Moreno et al. have also been found highly expressed in FFs from MII [31]. Moreover, both studies detected the higher expression levels of has-mir-451 in FFs in the early phases of oocytes maturation [30,32]. Also, miRNAs such as hsa-miR-139-3p were shown to be down-regulated in the FF from GV oocytes compared with MII oocytes [33].

**Figure 3:**
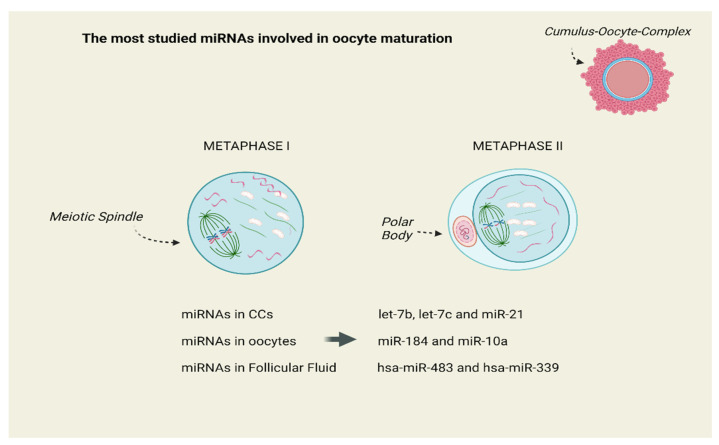
The most studied miRNAs expressed in Cumulus Cells (CCs), Oocytes and Follicular Fluid (FF), which regulate and control oocyte meiotic maturation. Created with BioRender.com on 4 May 2024.

The miRNAs of human let-7 family were the subject in a series of studies. Assou et al. studied the expression of miRNA in human MII oocytes and in CCs and they revealed that the most abundant miRNAs in CCs were let-7b, let-7c and miR-21, while miR-184 and miR-10a were mainly detected in oocytes [34]. Andrei et al. revealed that miR-21-5p, let-7a-5p and let-7f-5p miRNAs were significantly over-expressed in GCs and CCs. Hence, the authors proposed that differentially expressed miRNAs are involved in the regulation of steroidogenesis, apoptosis and proliferation of GCs [35]. According to Jenabi et al., miR-21 was down-regulated in women with female factor infertility [36]. Therefore, miR-21 may have a major developmental role in oocytes via suppression of genes, promoting apoptosis in CCs [37,38,39]. When Bartolucci et al. examined microRNA-21 in human oocytes, the cultured CCs treated with an mir-21-5p inhibitor showed an increased apoptotic rate followed by a marked PTEN expression (a gene that is well-known for inhibiting the AKT-dependent survival pathway in cumulus cells) [40]. Figure 3 presents the most studied miRNAs expressed in cumulus cells (CCs), oocytes and follicular fluid (FF), which regulate and control oocyte meiotic maturation.

Dysfunctions in ovary tissue may cause various miRNAs alterations and affect oocytes. The miR-548 members seem to have a crucial role in maintaining oocyte function. Oltean et al. reported that hsa-miR-548au-3p was down-regulated and hsa-miR-548ae-5p, hsa-miR-548t-3p and hsa-miR-548au-5p were up-regulated [41]. Zhang et al. examined 68 patients by collecting ovarian FF and detected 47 miRNAs, which were involved in oocyte quality. Among two groups (high and poor oocyte quality), seven miRNAs were up-regulated in the poor oocyte quality group [42]. The down-regulation of hsa-miR-513c-5p in the poor oocyte quality group is expected to affect oocyte quality and is related according to other studies in breast cancer and hormonal dysregulation [43]. The up-regulation of miR-505-3p in the group of poor oocyte quality is possibly related to infertility. Its over-expression in mice was associated with delayed ovary maturation and lower fertility [44]. However, Barragán and colleagues, by examining a total of 36 in vivo matured MII oocytes collected from 30 healthy women recruited for oocyte donation, found that five miRNAs such as hsa-miR-220b, miR-4262 and miR-1260a were increased in the older group (age 32 ± 2 years and high antral follicular count approx. 29 ± 7 follicles) [45].

The miR-320 family plays a pivotal role in embryo development [46]. Machtinger et al., by collecting FF from 40 women that underwent IVF treatment, studied 754 exmiRNAs expression. The authors identified 207 extracellular microRNAs (exmiRNAs); miR-30d-5p, miR-320b, miR-10b-3p, miR-1291, and miR-720 were the most detected. Four ex-miRNAs (miR-202-5p, miR-206, miR-16-1-3p, and miR-1244) had a higher level of expression in fertilized oocytes [47]. Those findings are in alignment with the surveys of Sang et al. and Moreno et al., who also documented the up-regulated expression of miR-720 detected in FF from mature oocytes [30,48]. Additionally, Zhang et al. showed that hsa-miR-320e was up-regulated in the poor oocyte quality group, proposing that this up-regulation might affect the proliferation of ovarian GCs, resulting in oocyte degradation [42]. Santonocito et al. collected FF and plasma from 15 women undergoing IVF and identified miR-10b, miR-29a, miR-99a, miR-125b, miR-132, miR-202, miR-212, and miR-874 to be highly expressed in granulosa or in cumulus cells. They also supported that miR-29a, miR-99a, miR-100, miR-132, miR-212, miR-214, miR-218, miR-508-3p, and miR-654-3p may have a regulatory role through molecular signaling pathways (WNT, MAPK, ErbB, and TGFβ) [49].

Angiogenesis triggered by hypoxia happening in aging follicles remains important. According to this, miRNAs play an essential role in the cellular feedback to hypoxia. Al-Edani et al. studied CC samples of older women (>37 years) and compared them with CC samples of younger (<30 years) and median (31–34 years) age. Authors showed that mir-425, mir-744, mir-146b, and Let-7d were expressed in younger and median age women, while mir-202 and Let-7e were expressed in the older women. All the aforementioned miRNAs are targeting hypoxia- and angiogenesis-related genes [50,51].

Finally, an altered expression of miRNAs like miR-145, miR-92 and miR-21 has been reported, which target ACVR1b and SMAD7, and inhibit the apoptosis of GCs [52,53,54]. On the contrary, miR-205 and let-7g promote the apoptosis of GCs and inhibit estrogen synthesis via CREB1 and MAPK3K1 signaling pathways [55]. Lastly, a recent study in GCs from women with diminished ovarian reserve found increased expression of miR-484, correlated with ovarian function and triggering of granulosa cells [56]. Supplementary Table S1 illustrates the design and main outcomes of all the included studies in the review.

## 4. Discussion

Oocyte maturation is a multiplex process consisting of a battery of biological mechanisms; a plethora of molecules and signaling pathways regulate that process. The rapid technological development such as genetic modification of animal models, molecular biology, and biochemistry helps the researchers to gain a better understanding of oocyte GV arrest, meiosis I resumption and miRNAs expression. Because the oocyte maturation is not only self-depended, but it is based on mutual feedback and physical contact with follicular granulosa cells, it is interesting to focalize on different molecular pathways involving oocytes, granulosa cells, and oocyte microenvironment.

**Figure 4:**
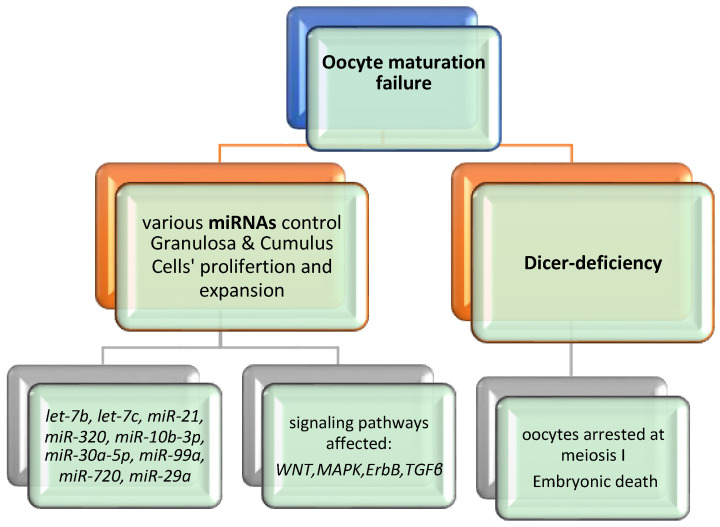
Overview of oocyte maturation failure.

Studying the miRNAs in reproductive medicine represents a great challenge as it highlights important molecular pathways in female reproductive tissues and embryonic development. Consequently, better knowledge may provide new biomarkers and more personalized approaches for fertility issues. According to Eppig et al. oocyte maturation is a process in which the oocyte undergoes different transformations from the prophase I to metaphase II (MII) through meiotic division, chromatin remodeling and organelle reorganization [57]. Additionally, it acquires a transcriptomic and proteomic content essential for the early stages of embryonic development [58]. Consequently, understanding the mechanisms of protein expression and mRNA differentiation, should be the main field of research regarding female oogenesis. Specific miRNA profiling studies propose that changes in the expression pattern of miRNAs may be detected during the growth and development of mammalian oocytes. For example, miR-21, let-7family, miR-27a and miR-322 and DICER are essential in oocyte development and many research groups have established a possible interaction between their expression and oogenesis. miRNAs seem to regulate essential mechanisms like apoptosis, meiosis, hormone secretion and intracellular molecular pathways [33,34,36]. Figure 4 illustrates an overview of oocyte maturation failure and the affected signaling pathways. 

The loss of Dicer is a crucial factor in oogenesis, oocyte maturation and embryogenesis [59]. The vital role of Dicer can be documented in the case of Dicer deficiency, resulting in embryonic death [60,61]. Moreover, the involvement of Dicer in oogenesis was proposed through conditional knockout experiments, in which oocytes lacking Dicer arrest at meiosis I [62,63]. Liu et al. confirmed the above theory and proposed that Dicer and miRNA were essential for spindle organization, meiotic maturation and consequently control of the quality of oocytes [64]. As the most abundant miRNAs in CCs are revealed to be let-7b, let-7c and miR-21, members of let-7 family and mir-21 seem to have an essential role in female reproduction due to their involvement in the regulation of steroidogenesis, in apoptosis and in the proliferation of GCs [34,35,36,39,40]. Table 1 provides a summary list of the most studied miRNAs and their involvement in oocyte development and maturation.

## 5. Conclusions 

The present study is the first to our knowledge to provide an overview on miRNA expression related to oocyte maturation. Even though the current review highlights the number of miRNAs known to interfere with oocyte-related proteins, it would also be of great interest to focus on the polymorphisms detected on the corresponding genes and their effect on female reproduction.

Finally, it should be noted that even though the cause of miRNA-differentiated expression is unclear, epigenetic changes, genetic mutations and oxidative stress can be possible factors, causing their dysregulation. It is of major importance to clarify the role and mechanism of each miRNA as understanding its action may develop new tools or markers and establish pioneer approaches for diagnosis and treatment of infertile couples and ovarian dysfunction.

## Figures and Tables

**Table 1:** Summary list of miRNAs and their involvement in oocyte development.

miRNA	Cell Type	Regulation	Regulatory Role	References
let-7a-5p	GCs and CCs	up	Proliferation of GCs and CCs	[35]
let-7b	CCs	up	Steroidogenesis Apoptosis and proliferation of GCs	[34]
let-7c	CCs	up	Steroidogenesis Apoptosis and proliferation of GCs	[34]
let-7f-5p	GCs and CCs	up	Proliferation of GCs and CCs	[35]
miR-21	CCs	up	Oocyte maturation Steroidogenesis Apoptosis and proliferation of GCs	[34,35,36,37,38,39,52,53,54]
miR-21	CCs	down	Female infertility	[36]
miR-21-5p	GCs and CCs	up	Apoptosis of GCs and CCs	[35]
hsa-miR-320e	GCs	up	Proliferation of GCs affecting Oocyte degradation	[42]
miR-99a	GCs and CCs	up	Poor oocyte quality	[31,49]

## Data Availability

Not applicable.

## Supplementary Materials

The following supporting information can be downloaded at: https://www.mdpi.com/article/10.3390/diseases12060121/s1, Table S1: Included studies in the review.
