# Supplementary material for: Oocyte Maturation and miRNAs: Studying a Complicate Interaction to Reveal Possible Biomarkers for Female Infertility
Source: Diseases. 2024 Jun 4;12(6):121. doi: 10.3390/diseases12060121 (PMC11202488; doi:10.3390/diseases12060121)
Supplement: Supplementary file 1 [file diseases-12-00121-s001.zip › diseases-2956683-supplementary.pdf]

Supplementary Table S1. Included studies in the review.

Study	Year	Study Design	Main Outcome
Tatemoto et al.[25]	2000	Porcine animal model	Cumulus cells protect oocytes against apoptosis induced by oxidative stress.
Dompe et al.[26]	2021	Review	Mural Granulosa Cells and Cumulus Cells are under the influence of various miRNAs regarding oocyte maturation.
Jagarlamudi et al.[27]	2012	Review	Transcriptional regulators (Foxo3, Foxl2, Figla, Lhx8, Nobox, Sohlh1 and Sohlh2) are associated with ovarian formation and folliculogenesis.
Da Silveira et al.[28]	2012	Comparative study with follicular fluid samples	Different miRNAs expression was observed between young and old study group.
Sirotkin et al.[29]	2010	Review	Micro RNAs are involved in both proliferation and apoptosis of ovarian granulose cells.
Moreno et al.[30]	2015	Observational Study in 30 women undergoing ICSI	hsa-miR-424 was differentially expressed in the follicular fluid of the advanced maternal age group.
Santonocito et al.[31]	2014	Case series of 15 women undergoing ICSI	- miR-29a, miR-99a, miR-100, miR-132, miR-212, miR-214, miR-218, miR-508-3p, and miR-654-3p were upregulated in follicular fluid and in exosomes and are associated with meiosis resumption.
Hou et al.[32]	2014	Animal model study	MiR-451 can be a potential biomarker for estrogenicity in the uterus.
Yang et al.[33]	2012	Research article	Mir-23a regulates apoptosis by downregulation of XIAP expression in ovarian granulosa cells.
Assou et al.[34]	2013	Case series in MII oocytes and cumulus cells from women undergoing IVF	- MIR184, MIR100 and MIR10A were over-expressed in MII oocytes.<br>- MIR29a, MIR30d, MIR21, MIR93, MIR320a, MIR125a and the LET7 family in the cumulus cells.
Andrei et al.[35]	2018	Research article	53 miRNAs were differentially expressed between Mural Granulosa Cells and Cumulus Cells
Jenabi et al.[36]	2023	Case series with 87 women	miR-21 plays an important role in oocyte development and is down-regulated in women with female factor infertility.
Almeida et al.[37]	2018	Review	Apoptosis of granulosa cells negatively affect pregnancy rates in ART.
Carletti et al.[38]	2010	Mice animal model	mir-21 has a physiologic role in tissue function.
Han et al.[39]	2017	Animal model	miR-21 is associated with suppression of Cumulus Cells apoptosis.
Bartolucci et al.[40]	2020	Case series in 25 patients	miRNA-21 in human cumulus cells affects the developmental potential of oocytes.
Oltean et al.[41]	2021	Review	Gene expression analysis indicated that endometriosis and ovarian cancer are associated with ARID1A, PI3KCA, PTEN, BRCA1, BRCA2, TP53 and KRAS genes mutations.
Zhang et al.[42]	2021	Case series with 68 women	- hsa-miR-505-3p was over-expressed in the poor oocyte quality group.<br>- hsa-miR-548ae-5p, hsa-miR-548t-3p, hsa-miR-548au-5p, and hsa-miR-548au-3p are associated with the maintenance of oocyte quality.<br>- hsa-miR-320e affects the proliferation of ovarian granulosa cells and oocyte degradation.
Muti et al.[43]	2018	Cohort Study/382 women	miR-513a-5p is upregulated in breast cancer
Zhou et al.[44]	2018	Mice animal model	miR-505-3p plays a role in the regulation of sexual maturation in mammals.
Barragán et al.[45]	2017	Case series / 36 MII oocytes	Age and ovarian reserve are associated with specific non-coding RNA profiles.
Feng et al.[46]	2015	Case series / 53 oocytes matched with	- miR-320 and miR-197 in FF affect embryonic developmental potential.

Machtinger et al.[47]	2017	53 follicular fluid samples<br>Case series / 40 women undergoing IVF	- Knockdown of miR-320 inhibits embryonic development.<br>exmiRNAs in the follicular fluid alter fertilization and day 3 embryo morphology.
Sang et al.[48]	2013	Study / FF samples	- miRNA-132 and miRNA-320 are expressed in women with polycystic ovary syndrome.<br>- miR-24, miR-132, miR-320, miR-222, miR-520c-3p, miRNA-193b, and miRNA-483-5p regulate hormonal secretion
Conti et al.[49]	2012	Review	LH affects the expression of *Areg* and *Ereg* miRNAs.
Al-Edani et al.[50]	2014	Study / Human CCs	Pathways associated with age are targets of specific miRNAs
Barragán et al.[51]	2023	Case series with 94 women	NOS2, HMOX1, and VEGFC expression are indicative for oocyte maturation and fertilization rate of oocytes.
Yan et al.[52]	2012	Mice animal model	miR-145 inhibits mouse granulosa cells proliferation.
Liu et al.[53]	2014	Porcine animal model	- miR-92a is over-expressed in healthy follicles and can inhibit granulosa cell apoptosis.<br>- miR-92a targets Smad7
Sinha et al.[54]	2017	Bovine animal model	miR-130b affects oocyte maturation and embryonic development.
Cao et al.[55]	2015	Porcine animal model	let-7g induces granulosa cell apoptosis by inhibiting the MAP3K1 gene.
Li et al.[56]	2022	Study/156 samples of human FF	miR-484 is associated with mitochondrial dysfunction, and granulosa cells apoptosis.
